# Cyclin A1 Modulates the Expression of Vascular Endothelial Growth Factor and Promotes Hormone-Dependent Growth and Angiogenesis of Breast Cancer

**DOI:** 10.1371/journal.pone.0072210

**Published:** 2013-08-08

**Authors:** Azharuddin Sajid Syed Khaja, Nishtman Dizeyi, Pradeep Kumar Kopparapu, Lola Anagnostaki, Pirkko Härkönen, Jenny Liao Persson

**Affiliations:** 1 Department of Clinical Sciences, Lund University, Malmö, Sweden; 2 Department of Laboratory Medicine, Lund University, Malmö, Sweden; 3 Department of Clinical Pathology, Skåne University Hospital, Malmö, Sweden; 4 Institute of Biomedicine, University of Turku, Turku, Finland; II Università di Napoli, Italy

## Abstract

Alterations in cellular pathways related to both endocrine and vascular endothelial growth factors (VEGF) may contribute to breast cancer progression. Inhibition of the elevated levels of these pathways is associated with clinical benefits. However, molecular mechanisms by which endocrine-related pathways and VEGF signalling cooperatively promote breast cancer progression remain poorly understood. In the present study, we show that the A-type cyclin, cyclin A1, known for its important role in the initiation of leukemia and prostate cancer metastasis, is highly expressed in primary breast cancer specimens and metastatic lesions, in contrasting to its barely detectable expression in normal human breast tissues. There is a statistically significant correlation between cyclin A1 and VEGF expression in breast cancer specimens from two patient cohorts (*p*<0.01). Induction of cyclin A1 overexpression in breast cancer cell line MCF-7 results in an enhanced invasiveness and a concomitant increase in VEGF expression. In addition, there is a formation of protein–protein complexes between cyclin A1 and estrogen receptor ER-α cyclin A1 overexpression increases ER-α expression in MCF-7 and T47D cells. In mouse tumor xenograft models in which mice were implanted with MCF-7 cells that overexpressed cyclin A1 or control vector, cyclin A1 overexpression results in an increase in tumor growth and angiogenesis, which is coincident with an enhanced expression of VEGF, VEGFR1 and ER-α Our findings unravel a novel role for cyclin A1 in growth and progression of breast cancer, and suggest that multiple cellular pathways, including cell cycle regulators, angiogenesis and estrogen receptor signalling, may cooperatively contribute to breast cancer progression.

## Introduction

Breast cancer accounts for 41% of all cancers in women in the United States alone and represents a major clinical challenge [1]. Despite a significant improvement in drug discovery and therapies for breast cancer during the past decades, yet no effective treatment regimens are available for patients with invasive and metastatic breast cancer. In addition, a large number of treated patients will experience disease recurrence, which often do not respond to therapy [2]. Thus, there is an urgent need to gain deeper understanding of cellular mechanisms underlying the progression and metastasis of breast cancer and to identify new targets for designing novel therapeutic drugs.

Deregulation of estrogen pathway is known to be a major cause of breast cancer. At least 70% of breast cancer display an elevated level of estrogen receptor-alpha (ER-α), and are therefore classified as ER-α positive subtypes [2]. ER-α mediates estrogen effects and promotes growth and survival of breast cancer cells, and is therefore a main target for anti-estrogen therapies [3]. Treatment of ER-α positive breast cancer with anti-estrogen therapy achieves the effects through interfering of drugs with ER-α action [2]. It is known that ER-α exerts its effect on the growth of tumor cells in cooperation with its co-factors that are required for cellular proliferation [2]. ER-α and its co-activator such as nuclear receptor co-activator 3 (NCOA3), form protein–protein complexes to activate the transcription of their target genes and therefore promote cellular proliferation [2].

Several molecules that are downstream targets of ER-α may also affect cellular sensitivity to estrogen stimulation. Vascular endothelial growth factor (VEGF) is expressed in normal breast and uterine cells and its expression is regulated by ER-α in response to estrogen stimuli [4–7]. VEGF, a key factor in angiogenesis has been shown to promote tumor growth, invasion and distant metastasis in several cancer types such as leukemia and breast cancer [8–10]. The autocrine and paracrine signalling mediated by VEGF and its receptors contribute to the increased proliferation and survival of breast cancer cells [11–13]. Estrogen treatment induces VEGF expression only in ER-α positive MCF-7 cells, but not in ER-α negative MDA-MB-231 cells [5,14,15], suggesting that VEGF expression is regulated by an ER-dependent mechanism. Studies in breast cancer patients show that VEGF expression in breast cancer cells correlates with impaired response to therapy and poor patient outcomes [4,16,17]. Targeted therapy using Bevacizumab, a monoclonal antibody to neutralize VEGF is widely used to inhibit angiogenesis of tumors, and has shown beneficial effect for treatment of cancers of colorectal, renal cell and brain [18–20]. Thus, better understanding of the cellular mechanisms underlying the functional relationship between ER-α and VEGF is of importance to design novel therapies to co-inhibit endocrine and angiogenesis pathways for treatment of invasive breast cancer.

Cell cycle regulatory pathway plays an important role in estrogen-dependent and independent growth of breast cancer cells [2]. The anti-estrogen therapy is aimed to inhibit proliferation and survival of breast cancer cells, and its effect can be in part achieved by inhibiting the expression and activities of cell cycle regulators [2]. However, it remains unclear whether cell cycle pathways may be functionally linked to or overlap with both estrogenic and VEGF pathways. Cyclin A1 has been previously shown to play an important role in tumorigenesis. In normal tissues, cyclin A1 expression has mainly been detected in testes, hematopoietic cells and brain [21–24]. Only low level of cyclin A1 transcript is observed in the developing mammary gland, but its level becomes undetectable in adult gland during pregnancy [25]. In contrast to its low expression in the majority of normal tissues, elevated level of cyclin A1 is frequently observed in various types of cancers including prostate cancer, leukemia, testicular cancer, ovarian cancer and lung cancer [26–31]. Targeted overexpression of cyclin A1 in early myeloid cells initiated acute myeloid leukemia in transgenic mice [32]. We have previously shown that tumors from patients with advanced prostate cancer and distant metastases exhibited high level of cyclin A1 expression [33]. Cyclin A1 is able to mediate the effect of PI3K/AKT to promote survival of prostate cancer cells in the presence of high level of cytokine, interleukin-6 [34]. Moreover, cyclin A1 promotes invasion and metastasis of prostate cancer cells by increasing the expression of VEGF and matrix metalloproteinases (MMPs) [33]. High level of cyclin A1 mRNA expression has also been observed in various types of breast cancer cell lines [25]. A homeobox protein Six1 promotes oncogenic transformation of mammary epithelial cells through cyclin A1-mediated actions [35].

In the present study, we tested our hypothesis on that cyclin A1 overexpression may promote growth and invasion of breast cancer cells. Our data showed that induction of cyclin A1 overexpression in breast cancer cell line MCF-7 promoted cancer cell invasion and increased the expression of VEGF and ER-α. Using xenograft mouse models in which mice were implanted with MCF-7 xenograft tumor cells, we showed that MCF-7 xenograft tumors that overexpressed cyclin A1 grow into larger size than the tumors expressed control vectors. Our findings suggest that cyclin A1 associated pathways play important roles in breast cancer progression.

## Methods

### Tissue Specimens, Tissue Microarrays and cDNA microarray databases

The first set of Tissue microarray (TMA) that consists of the selected areas of primary breast tumors from 94 patients in duplicates with 1.0 mm in diameter was constructed at Department of Pathology, Umeå University and University Hospital in Umeå, Sweden. This TMA was used and described previously [36,37]. The second TMA that contains normal breast adjacent to tumors, primary tumors and metastatic lesions in lymph node (84 breast cancer specimens) from 48 patients was obtained from Pantomics Inc. (http://www.pantomics.com/, Pantomics, Inc., Richmond, CA). Cyclin A1 mRNA and VEGF expression data was obtained from the dataset in the Gene Expression Omnibus (GEO) database at the National Center for Biotechnology Information (NCBI) website. The dataset GEO accession number: GSD1096 was obtained by performing Affymetrix U133A array platform using human normal tissues from 36 organs (Figure S1) as previously described [38]. The study was approved by the Ethics Committee, Lund University and the Helsinki Declaration of Human Rights was strictly observed.

### Antibodies

The following antibodies were used in this study: For cyclin A1, polyclonal anti-cyclin A1, (US Biological, and Swampscott, MA); monoclonal anti-cyclin A1, (BD Pharmingen, and San Diego, CA), or polyclonal anti-cyclin A1 (Santa Cruz Biotechnology, Santa Cruz, CA) were used. Polyclonal human anti-CD31 and CDK1 (BD Pharmingen), anti-β-actin, anti-VEGF, anti-VEGF-R1 and VEGF-R2 (Santa Cruz Biotechnology, CA), anti-ER-α, Ki67 (Dako, Golstrup, Denmark) and anti-cyclin D1 (Cell Signaling Technology Inc, Danvers, MA).

### Immunohistochemistry analysis

Immunohistochemistry on tumor tissue arrays was performed as previously described [26]. The staining procedure was performed using a semiautomatic staining machine (Ventana ES, Ventana Inc., Tucson, AZ). The sections were viewed with an Olympus BX51 microscope at magnification of 20x or 40x. The specimens were evaluated by four different specialists; two of them are specialists in pathology. The staining intensity was scored as 0 (negative), 1 (weakly positive or positive), 2 (moderate positive), 3 (strongly or very strongly positive) using an arbitrary semi-quantitative scale. The staining for both cytoplasmic and nuclear compartments was considered. The slides were scanned and microphotographs were taken by using the scanner (ScanscopeCS, Aperio, Vista, CA). For immunohistochemistry of mouse organs, tissues were fixed in 4% paraformaldehyde for 24 hours and embedded in paraffin. For histology analysis, the sections were stained with hematoxylin-eosin (H&E) and were subjected to analysis under Olympus BX51 microscopy.

### Cell lines

Six breast cancer cell lines: T47D, BT549, MCF-7, MDA-MB-468, MDA-MB-231 and Cama-1 were purchased from American Type Culture Collection (ATCC, Manassas, VA). The cells were maintained in RPMI1640 medium supplemented with 10% serum. The major clinical and molecular properties of each cell line are listed in Table 1.

**Table 1:** Characteristics of different breast cancer cell lines.

**Cell lines**	**Cell Types**	**ER**	**P53**	**pRb**	**Receptors expressed**
T47D	DC (pe)	+	Mutated	+	AR, PR [40,46]
MCF7	A (pe)	+	WT	+	Progesterone receptor [40]
MDA 468	A	-	Mutated	-	EGFR [47], TGF-α [48]
MDA 231	A (pe)	-	Mutated	+	EGF, EGFR [47,49], TGF-α [49]
Cama-1	A (pe)	+	Mutated	+	AR [50]

### Stable and Transient Transfection

For transient transfection, pCMS-EGFP control vector as “pCMS-EGFP” and pCMS-EGFP containing a 1.8 kb full-length human cyclin A1 cDNA as “cyclin A1-pCMS-EGFP” were used and were described previously [33]. Transient transfection was performed using the electroporation system with Nucleofection kit (AmaxaBiosystems, Gaithersburg, MD) according to the manufacturer’s instructions. For stable transfection, MCF-7 cells were transfected with control pcDNA vector and pcDNA vector containing full-length human cyclin A1 cDNA using the electroporation system with Nucleofection kit (Amaxa Biosystems). The cyclin A1-pcDNA vector was kindly provided by Dr. Carsten Muller-Tidow (Department of Medicine, University of Munster, Munster, Germany) and was described previously [21]. 2–5 µg vectors were used in transfection experiments. Cells overexpressing cyclin A1-pcDNA or pcDNA vector were selected by culturing cells in medium containing G418 antiboitics.

### RNA isolation and RT–PCR

RNA was isolated from breast cancer cell lines as previously described [26]. RNA was reversely transcribed to cDNA following the supplier’s protocol (Invitrogen). The following primers were used: VEGF – forward: 5' - CTG CTG TCT TGG GTG CAT TGG –3'; VEGF – reverse: 5' - CAC CGC CTC GGC TTG TCA CAT –3' ; Cyclin A1 - forward: 5' – GTC AGA GAG GGG ATG GCA T –3'; Cyclin A1 - reverse: 5' – CCA GTC CAC CAG AAT CGT G –3' ; ER-α - forward: 5' – GTG GGA ATG ATG AAA GGT GG –3'; ER-α - reverse: 5' – TCC AGA GAC TTC AGG GTG CT –3'; GAPDH - forward: 5' – AAC AGC GAC ACC CAC TCC TC –3' ; GAPDH – reverse: 5' – GGA GGG GAG ATT CAG TGT GGT –3'; β-actin - forward: 5' – GCT CGT CGT CGA CAA CGG CTC –3'; β-actin – reverse: 5' – CAA ACA TGA TCT GGG TCA TCT TCT C –3'

The PCR conditions were: denature the DNA at 95° C for 5 min, followed by denaturing at 94° C for 1 min, then annealing at 55° C for 1 min, and extension at 70° C for 1 min with 18 cycles using only VEGF or cyclin A1 primers, then with another 17 cycles after the addition of GAPDH primers and finally with the incubation procedure at 70° C for 10 min. Semin-quantifications were performed using densitometric analysis program Quality one-4.6.2 (Bio-Rad, Hercules, CA).

### *In vivo* Animal Studies

Athymic NMRI female nude mice aged 8–12 weeks from Taconic Europe (Lille Skensved, Denmark) were used in the experiments. 17-β-estradiol pellets (Innovative Research of America, Sarasota, FL) were either implanted into the mice at dose 0.72 mg/per pellet or were injected intra-peritoneal into mice at dose 5 mg/kg weigh 3 times a week. Two sets of tumor xenograft mouse models were established. In the first model, MCF-7 cells were transiently transfected with cyclin A1-pCMS-EGFP or pCMS-EGFP control vector. Subsequently, 1× 10^6^ tumor cells were implanted subcutaneously into the lateral area of the neck of control mice “pCMS-EGFP mice” (n=4) and of experiment mice “cyclin A1-pCMS-EGFP mice” (n=5) at time of cell injection. One mouse from control group and one from experiment group were excluded in tumor growth curves due to the absence of appearance of tumors in these mice. In the second model, MCF-7 cells stably expressing cyclin A1-pcDNA or pcDNA vectors were implanted into the lateral area of the neck of mice (n= 3 per group). Upon tumor initiation, aliquots of 4 × 10^6^ viable cells per mouse suspended in 50 µl sterile PBS mixed with 50 µl Matrigel (BD Biosciences, Bedford, MA) were used. All animal experiments were approved and the regulations of the Animal Welfare at Lund University were strictly followed. At the end of experiments, mice were sacrificed under anesthesia using a mix of isofluorane and oxygen delivered by mask. Tumors were formed between 7 and 14 days post injection. Tumor diameters were measured using calipers, and volumes were calculated using the equation (a*x*b^^2^^ **/2) [39], where *a* and *b* represent the larger and smaller diameters, respectively. Tumor tissues were removed from mice and were used for histology and immunohistochemical analysis were fixed in 4% paraformaldehyde and embedded in paraffin. The sections were stained with H&E and were subjected to analysis under microscopy. At least two sections per tumor were determined and analyzed.

### Analysis of Angiogenesis

For tumor angiogenesis, tumor sections were stained with antibody against CD31. The regions of high vascular density within the tumors were examined. The number of CD31-positive pixels per microscopic field was scored. At least two sections per tumor and 3 views per section were determined.

### Immunoblotting and Immunoprecipitation Assays

Immunoblot analysis was performed as previously described [26]. For immunoprecipitation, a total of 500 μg of protein lysates was incubated with 3-5 (mu)g anti-ER-α or anti-cyclin A1 antibodies or equal amounts of mouse IgG or rabbit IgG as controls together with protein A or G beads (Pierce, Rockford, IL, USA) at 4° C overnight. Total protein lysates or lysates containing immune-complexes were separated on 12% sodium dodecyl sulfate (SDS)–polyacrylamide gels and were subsequently transferred to nitrocellulose membrane Hybond ECL (Amersham Pharmacia Biotech, Buckinghamshire, UK). The membranes were probed with appropriate primary antibodies, and then incubated with horseradish peroxidase–conjugated secondary antibodies (Amersham Life Sciences, Alesbury, UK) and visualized using the Enhanced ChemiLuminescence detection system (ECL). Densitometric analysis on blots was performed by Quality one-4.6.2 (Bio-Rad, Hercules, CA).

### Detection of VEGF Expression by Enzyme-Linked Immunosorbent Assay

The enzyme-linked immunosorbent assay (ELISA) for the detection of human VEGF in lysates from breast cancer cell lines was performed as previously described [26]. Briefly, lysates from cells transfected with cyclin A1-pCMS-EGFP or with pCMS-EGFP control vector were diluted to the final concentration of 1 mg/ml. 100 µl of lysate was applied for measuring human VEGF using the VEGF ELISA kit (R&D Systems, Minneapolis, MN).

### Estrogen stimulation and tamoxifen treatment

For estrogen treatment, cells were maintained in 10% charcoal stripped medium (CSS) (Gibco, Life Technologies Corporation, Stockholm, Sweden) for 24 hr prior to treatment with 17-β-estradiol at 10 nM (Sigma-Aldrich Inc, Stockholm, Sweden). The cells were cultured for additional 48 hr before harvests. For tamoxifen treatment, cells were maintained in 10% charcoal stripped medium (CSS) for 24 hr prior to treatment with 200 nM tamoxifen (Sigma-Aldrich Inc, Stockholm, Sweden). For combination treatment using 17-β-estradiol and tamoxifen, cells were pretreated with 17-β-estradiol for 4 hr and tamoxifen was then added to the culture. The cells were cultured for 48 hr before harvests.

### Invasion Assay

The invasiveness of cells overexpressing cyclin A1 was measured using Matrigel-coated Boyden trans-well chambers (Millipore Corporation, Billerica, MA) according to the manufacturer’s protocol. Briefly, the MCF-7 or MDA-MB-231 cells were transfected with either cyclin A1-pCMS-EGFP or pCMS-EGFP vectors. Cells were seeded onto the membrane of the upper chamber at a concentration of 3×10^4^ in 400 μL medium. RPMI-1640 medium containing 10% FBS was added to the lower chamber. Cells that passed through the Matrigel-coated membrane to the lower chamber were stained with crystal violet and were then dissolved in 10% acetic acid. The absorbance of the stained cells was measured on a Chameleon (Hidex, Turko, Finland) ELISA plate reader.

### Cell Cycle Analysis

MCF-7, MDA-MB-231, T47D cells were transfected with either cyclin A1-pCMS-EGFP or pCMS-EGFP vectors and were maintained in RPMI 1640 containing 0.1% FBS for 48 hours as previously described [33]. Cells were then washed twice with 1x PBS containing 10% FCS, resuspended and incubated in 1 x PBS buffer containing 5 µg/ml propidium iodide (Sigma), 100 mM sodium citrate, pH 7.3 and 0.05 mg RNase A (Sigma) for 20 min at 4 °C. The cell fluorescence was measured in a FACS Calibur cytofluorometer (Becton Dickinson, Franklin Lakes, NJ) and analyzed using CELL Quest software (Becton Dickinson).

### Statistical Analysis

Possible pair-wise correlations between the groups were analyzed using the Spearman Rank Correlation Test. The statistical significance of differences between groups was evaluated by paired Wilcoxon’s rank sum test and Student’s *t* test. All outcome variables are presented as the mean values with 95% confidence intervals (CIs). For measurement of tumor growth, two-way repeated analysis of variance was performed using ANOVA test. All data are representative for two to three independent experiments. All statistical tests were two-sided and P values less than 0.05 were considered to be statistical significant.

## Results

### Elevated level of cyclin A1 significantly correlated with VEGF expression in tumor specimens from breast cancer patients

**Figure 1:**
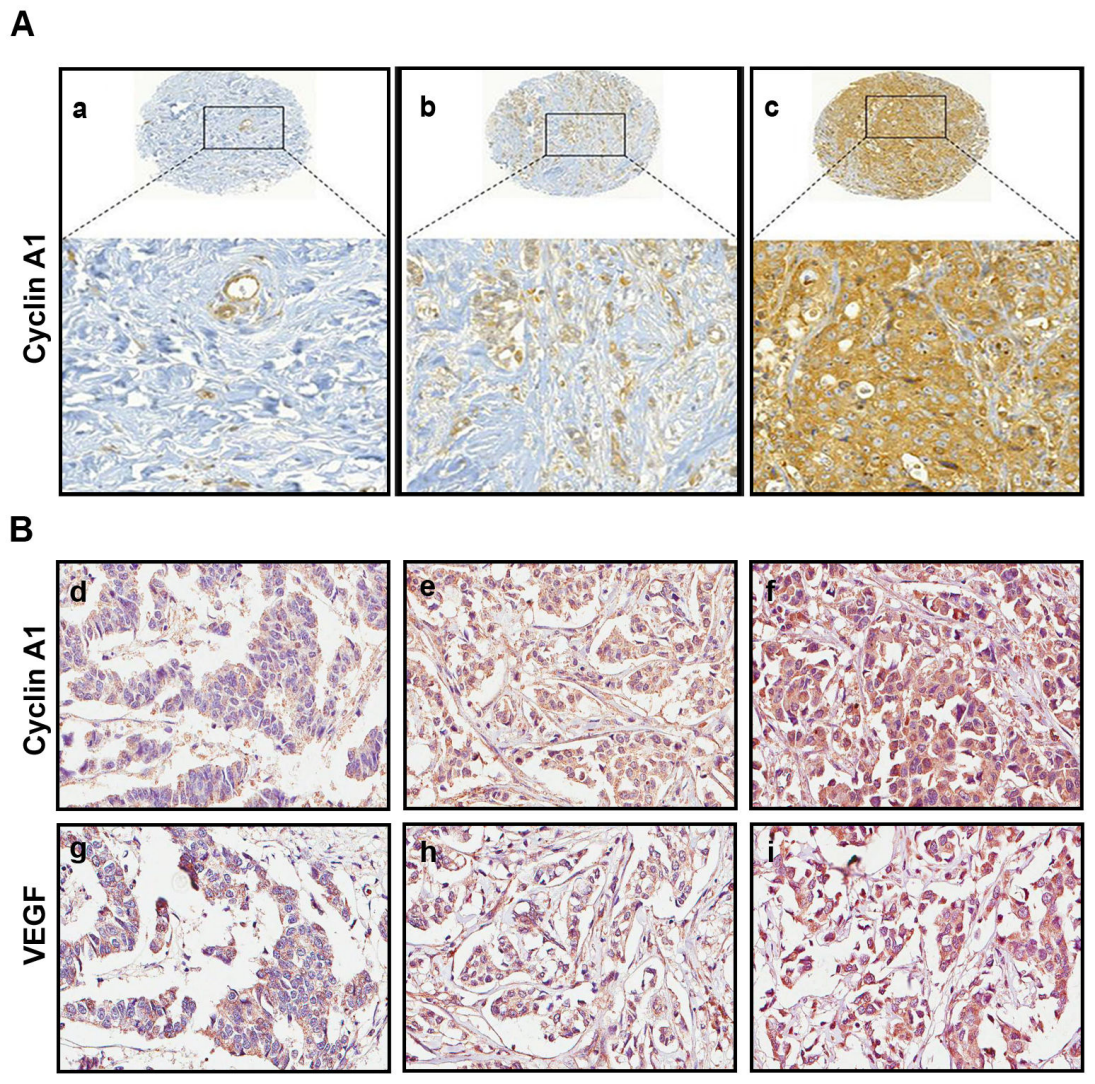
Evaluation of the expression of cyclin A1 and vascular endothelial growth factor (VEGF) in breast cancer specimens. (A) Primary breast cancer specimens from TMA1 were immunostained with antibodies against cyclin A1. Representative microphotographs of TMA cores (upper panels) and the enlarged area (lower panels) showing cyclin A1 staining intensities from weak (a), moderate (b) to high (c) levels are presented. (B) Representative microphotographs show the staining intensity of cyclin A1, weak (d), moderate (e) to high (f) and VEGF, weak (g), moderate (h) to high (i) in primary breast cancer specimens from TMA2.

To investigate whether cyclin A1 expression may be altered in patients with breast cancer, we first evaluated *cyclin A1* mRNA expression in normal human breast tissues using cDNA microarray expression profiles from public databases as previously described [38]. Among a panel of normal human tissues assessed (n=36), *cyclin A1* mRNA expression was very low in normal breast and in the majority of normal tissues from various types of organs, in contrast to its pronounced high level of expression in testes (Figure S1A). To search for a link between cyclin A1 and VEGF expression, we also examined *vegf* mRNA expression using the same databases. Similar to *cyclin A1, vegf* mRNA expression was also relatively low in normal breast tissue (Figure S1B) compared with other types of normal tissues examined. To obtain information on cyclin A1 expression in breast cancer specimens, we examined cyclin A1 expression in two sets of TMAs that contained tissues from clinically and pathologically annotated breast cancer cases. One set of TMAs contained primary breast cancer specimens from a 94-patient cohort as described previously [36,37], whereas another set of TMAs contained tissues from a 48-patient cohort, in which paired non-malignant and malignant specimens from 12 patients, along with paired primary and metastasis lesions from 36 patients were included. Immunohistochemical analysis showed that level of cyclin A1 expression was elevated in the majority breast cancer specimens (89%) in a 94-patient cohort (Table S1) and 79% in a 48-patient cohort (Table S2), as shown in the representative images (Figure 1A and B). Next, we examined VEGF expression in breast cancer specimens from the same TMAs as mentioned above. VEGF expression was similar to what was observed for cyclin A1 in breast cancer specimens (Figure 1B) (Table S2). We therefore assessed whether cyclin A1 expression may correlate with VEGF expression in the two breast cancer cohorts. We observed a statistically significant correlation between cyclin A1 and VEGF in cancer specimens from the two patient cohorts (In the 94-patient cohort: r^2^=0.362, *p*<0.01; and in the 48-patient cohort: r^2^=0.288, *p*<0.01) (Table 2) (Figure 1B). Cyclin A1 expression also correlated with a proliferation marker Ki67 in breast cancer specimens from the 94-patient cohort (r^2^=0.255 *p*<0.05) (Table 2). This data show that cyclin A1 is overexpressed in malignant breast tumors and its expression correlates with VEGF and Ki67 expression which are the indictors for tumor growth and progression.

**Table 2:** Correlation coefficients between levels of expression of cyclin A1, VEGF and Ki67 in breast cancer specimens.

Correlation Coefficient (*r* ^*2*^) ***Index***						
***TMA1***				***TMA2***		
*r* ^*2*^	*Cyclin A1*	*VEGF*	*Ki67*	*Cyclin A1*	*VEGF*	*Ki67*
Cyclin A1	-----	0.362^**^	0.255^*^	-----	0.288^**^	0.117
VEGF	0.362^**^	-----	0.124	0.288^**^	-----	0.282^*^

TMA1 (n=94) and TMA2 (n=48 primary cancer speciments+36 metastatic lesions) was determined using Spearman’s test. p<0.01 is indicated as “^**^”, and p<0.05 is indicated by “^*^”. Two-tailed test was applied to evaluate the level of significance.

### Correlation between cyclin A1 and VEGF expression and invasion and metastatic breast cancer cells

**Figure 2:**
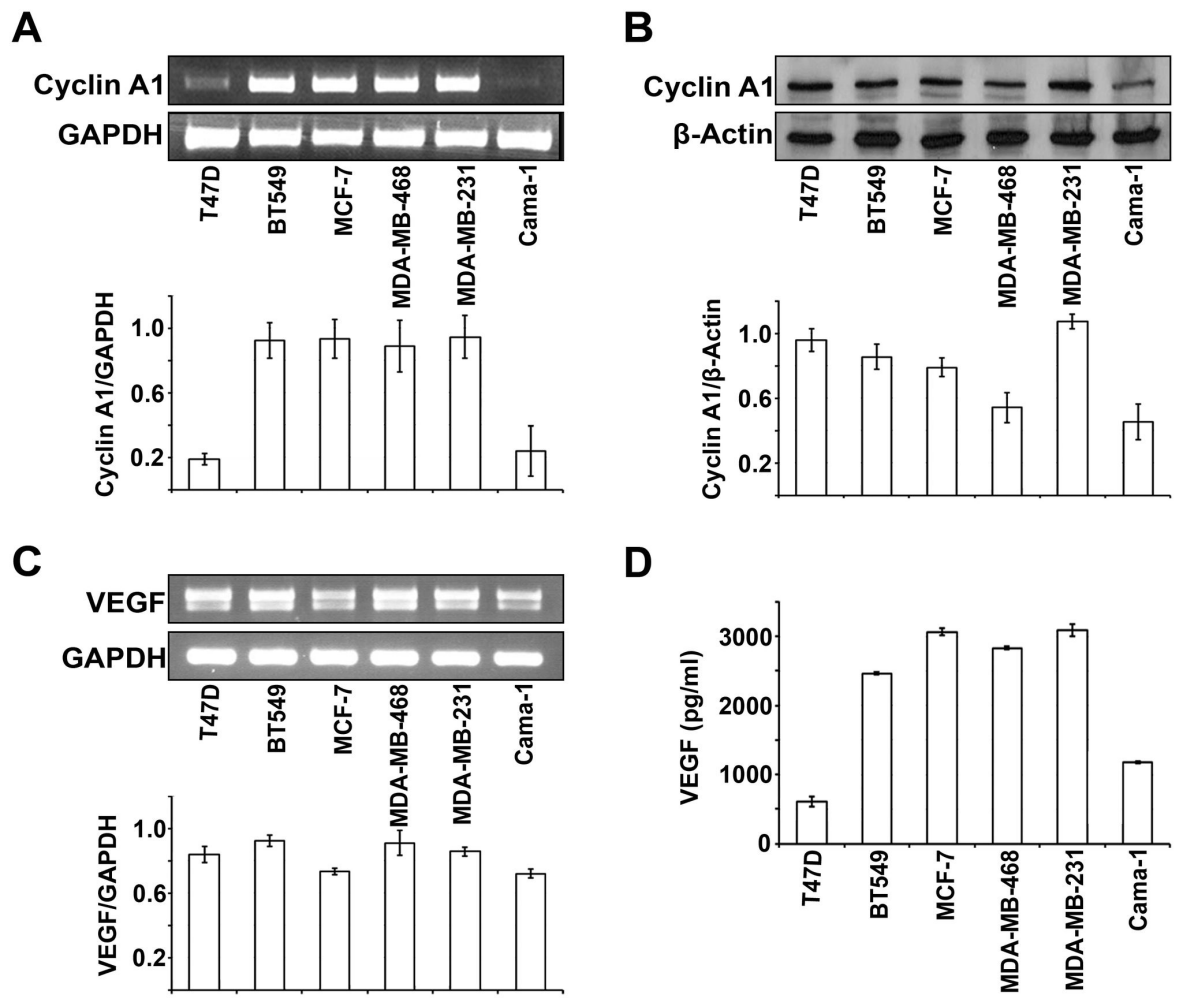
Evaluation of endogenous cyclin A1 and VEGF expression in breast cancer cell lines. (A) Cyclin A1 mRNA levels are assessed in T47D, BT549, MCF-7, MDA-MB-468, MDA-MB231 and Cama-1 cell lines by semiquantitative RT-PCR and a representative picture is shown (upper panel). Quantification of cyclin A1 mRNA level is shown and mean ± SD represents three independent experiments (lower panel). (B) Western blot analysis of levels of cyclin A1 protein in the cell lines as indicated. A representative picture is shown (upper panel). Quantification of cyclin A1 protein level is shown and mean ± SD represents three independent experiments (lower panel). (C) VEGF mRNA levels are assessed in the indicated cell lines by semiquantitative RT-PCR and a representative picture is shown (upper panel). Quantification of cyclin A1 protein level is shown and mean ± SD represents three independent experiments (lower panel). (D) ELISA assay of VEGF secretion in breast cancer cell lines as indicated. Mean ± SD represents three independent experiments.

**Figure 3:**
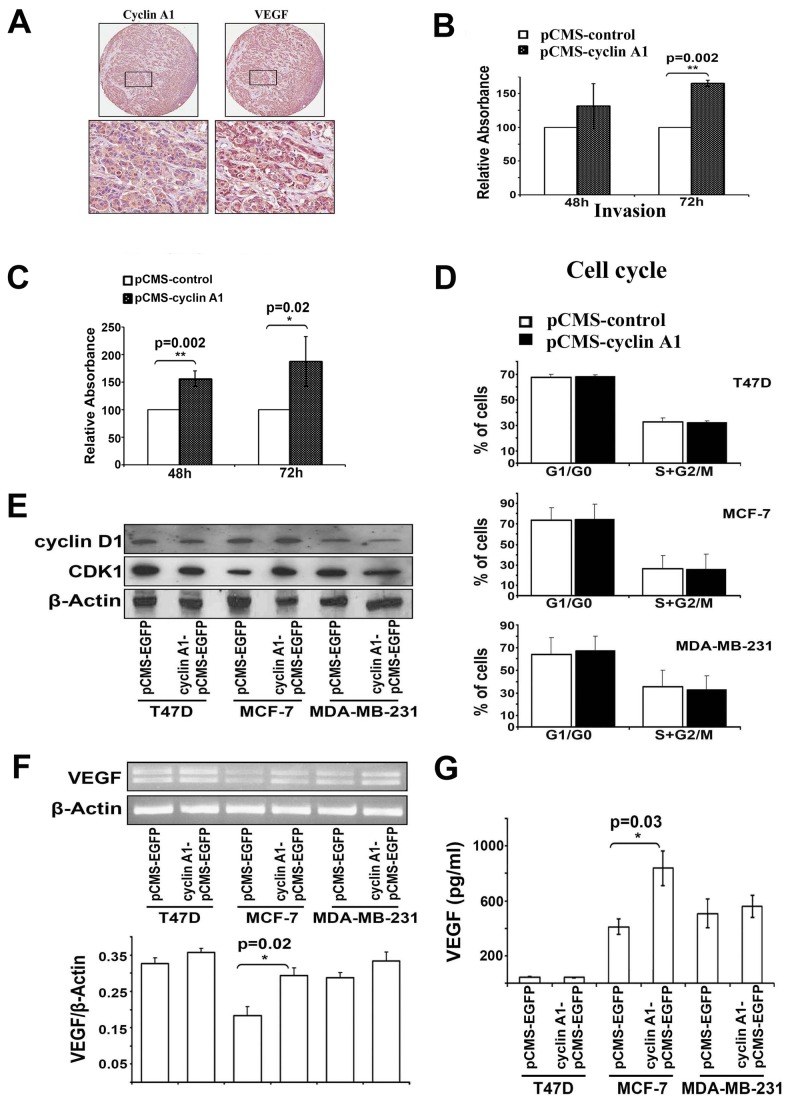
Effect of cyclin A1 expression on tumor invasion is associated with its effect on VEGF expression in MCF-7 cells. (A) Evaluation of cyclin A1 and VEGF expression in metastatic lesions from lymph nodes from patients with breast cancer metastasis using immunohistochemical analysis. Representative pictures show the cancer cells are strongly positive to cyclin A1 and VEGF expression. Upper panels represent cores at 20x magnificantion and lower panels represent the higher magnification (40x) of the selected areas. (B) MCF-7 cells that were transfected with cyclin A1pCMS-EGFP or pCMS-EGFP vectors were applied on the Matrigel-coated invasion chamber and were assessed after 48 or 72 hours. Data in graphs are the mean ± SD represents two independent experiments, each performed in duplicates. P value is indicated. (C) MDA-MB-231 cells transfected with cyclin A1pCMS-EGFP or pCMS-EGFP were applied on the Matrigel-coated invasion chamber and were analyzed after 48 or 72 hours. Data in graphs are the mean of two independent experiments, each performed in duplicate, p=0.002 for 48 h and p=0.02 for 72 h. (D) Cell cycle distribution of the cells that were transfected with cyclin A1pCMS-EGFP or pCMS-EGFP. Data in graphs are the mean ± SD represents three independent experiments from flow cytometry analysis. The percentage of cells at onset of each cell cycle phase is indicated. (E) Western blot analysis shows the levels of cyclin D1 and CDK1 protein expression in the cells that were transfected with cyclin A1pCMS-EGFP or pCMS-EGFP. (F) Representative picture shows the VEGF mRNA expression in the cells transfected with cyclin A1pCMS-EGFP or pCMS-EGFP (upper panel). Quantification of VEGF mRNA level in the samples is indicated. P value is shown (lower panel). (G) ELISA assay of VEGF secretion in the cells transfected with cyclin A1pCMS-EGFP or pCMS-EGFP. Mean ± SD represents three independent experiments (lower panel). Breast cancer cell lines used for these studies are T47D, MCF-7 and MDA-MB231 as indicated.

To investigate whether cyclin A1 may be involved in breast cancer progression and may be linked to VEGF pathways, we examined cyclin A1 and VEGF expression in various types of breast cancer cell lines (n=6) (Table 1). Both cyclin A1 mRNA and protein were highly expressed in BT549, MCF-7, MDA-MB-468 and MDA-MB-231 cells as measured and quantified by RT-PCR and immunoblot analyses (Figure 2A, 2B). High level of VEGF mRNA and protein expression was also observed in these cell lines (Figure 2C and 2D). Only T47D and Cama-1 cells displayed relatively low level of cyclin A1 mRNA and VEGF protein expression (Figure 2D). MDA-MB-231 cells that represented most aggressive type of breast cancer cells showed highest level of cyclin A1 protein expression, while MCF-7 cells that represented non-invasive and ER-α positive breast cancer displayed relatively low cyclin A1 expression (Figure 2B). To investigate whether expression of cyclin A1 and VEGF may be linked to breast cancer progression, we examined cyclin A1 and VEGF expression in breast cancer metastatic lesions. Primary breast cancer specimens and the corresponding metastatic lesions from lymph nodes of 36 patients were used for immunohistochemical analysis. Virtually all breast cancer metastatic lesions examined showed high level of cyclin A1 and VEGF expression (Table S3) (Figure 3A). These data suggest that cyclin A1 and VEGF are likely linked to the clinical progress of breast cancer.

To investigate whether cyclin A1 together with VEGF may play an important role in breast cancer progression and invasion, we performed functional analysis by employing three different breast cancer lines: MCF-7, MDA-MB-231 and T47D. As shown in Table 1, MCF-7 cells are the non-metastatic breast cancer cells and express ER-α while MDA-MB-231 cells have metastatic potentials and lack the expression of ER-α [40], and T47D is ER-α positive cell line but express low level of endogenous VEGF and cyclin A1. To this end, we introduced cyclin A1 overexpression by transfecting the cells with cyclin A1-pCMS-EGFP or pCMS-EGFP vectors. Overexpression of cyclin A1 in these three cell lines was achieved and confirmed by RT-PCR analysis (Figure S2). We first subjected MCF-7 cells and MDA-MB-231 cells transfected with cyclin A1-pCMS-EGFP or pCMS-EGFP vectors to invasion assays. Cells that migrated through the matrigel-coated boyden chambers were harvested after 48 hours and 72 hours, and the invasion rate of these cells were measured. MCF-7 cells transfected with cyclin A1-pCMS-EGFP vector had a significantly higher invasion rate compared with MCF-7 cells transfected with pCMS-EGFP vector at 72 hour-time point (*p*=0.002) (Figure 3B). Similarly, MDA-MB-231 cells transfected with cyclin A1-pCMS-EGFP vector are significantly more invasive than the cells transfected with pCMS-EGFP vector (For 48 hour-time point*, p*=0.002; For 72 hour-time point, *p*=0.02) (Figure 3C). Next, we examined the effect of cyclin A1 overexpression on cell cycle profiles in MCF-7, MDA-MB-231 and T47D cells. Flow cytometry analysis of cell cycle distribution revealed that induction of cyclin A1 overexpression in MCF-7, MDA-MB231 and T47D cells had no significant effect on cell cycle profiles (Figure 3D). Consistent with these findings, cyclin D1 protein expression was not influenced by cyclin A1 overexpression in these three breast cancer cell lines (Figure 3E). CDK1 expression was increased in MCF-7 cells that overexpressed cyclin A1 (Figure 3E). As CDK1 is a major kinase partner to cyclin A1, our data show that altered expression of cyclin A1 also leads to an alteration in CDK1 expression in breast cancer cells.

Next, we asked whether VEGF may be involved in mediating the invasive behaviour of breast cancer cells that overexpress cyclin A1. To this end, we examined VEGF mRNA and protein expression in MCF-7, MDA-MB-231 and T47D cells, which were transfected with cyclin A1-pCMS-EGFP or pCMS-EGFP vectors. VEGF mRNA and protein expression significantly increased in MCF-7 cells transfected with cyclin A1-pCMS-EGFP vector compared with the controls (For VEGF mRNA, *p*=0.02; for VEGF protein *p*=0.03) (Figure 3F and G). This suggests that overexpression of cyclin A1 results in an induction of VEGF expression in MCF-7 cells. However, VEGF mRNA and protein expression remained similar between MDA-MB-231 cells transfected with cyclin A1-pCMS-EGFP and the cells transfected with pCMS-EGFP (Figure 3F and G). Our data suggest that cyclin A1 influences VEGF expression in ER-α positive MCF-7 cells but not in MDA-MB-231 cells that lack ER-α expression.

### Cyclin A1 expression positively correlated with VEGF

**Figure 4:**
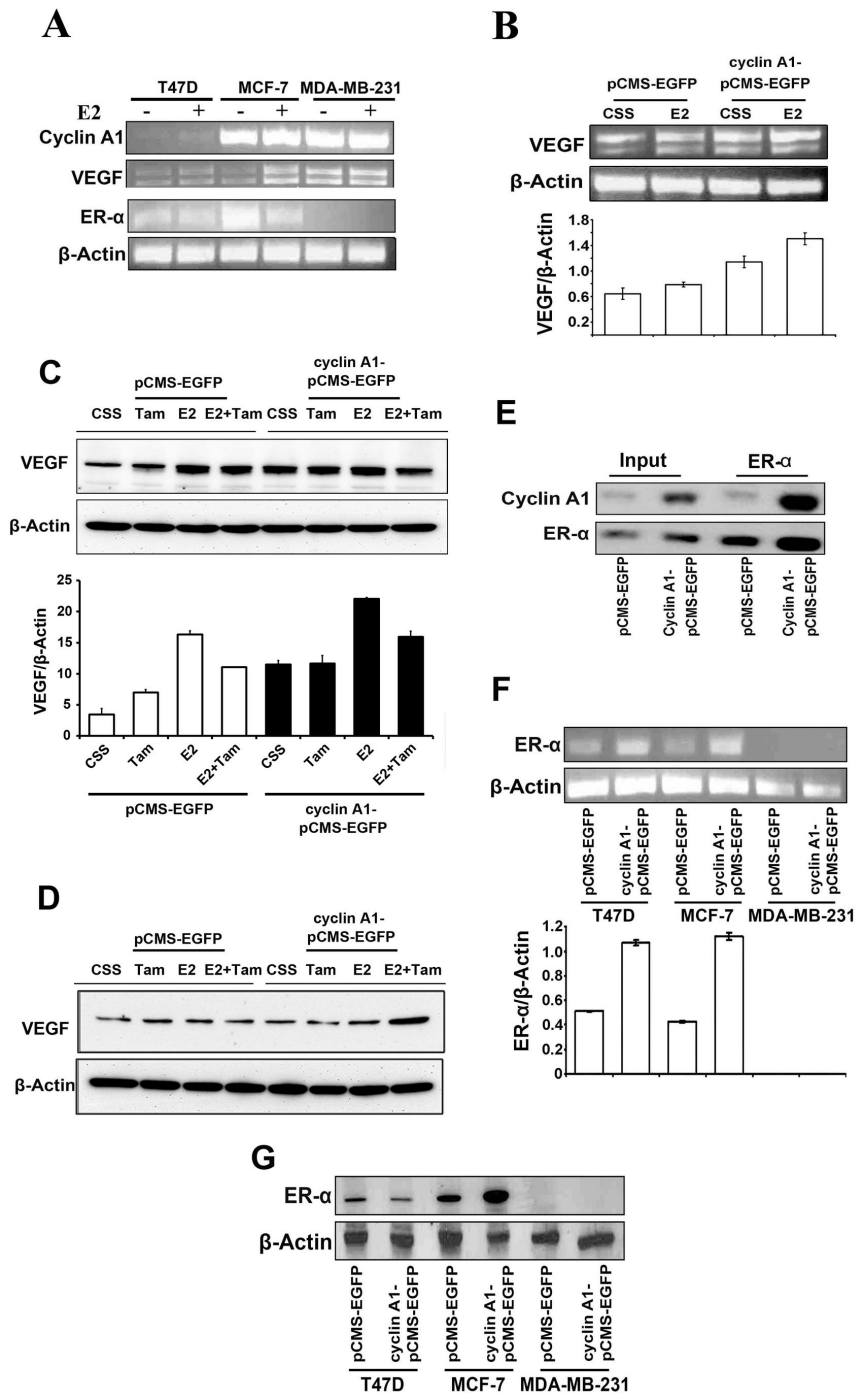
Evaluation of the association between cyclin A1 and ER-α estrogen signaling and the regulation of VEGF expression. (A) -Evaluation of the effect of estrogen on mRNA expression of cyclin A1, VEGF and ER-α, in T47D, MCF-7 and MDA-MB231 cells using semi-quantitative RT-PCR analysis. The three cell lines mentioned above were cultured in charcoal stripped medium (CSS) or in CSS medium containing E2 at 5 µM for additional 48 hours as indicated. (B) The effect of E2 treatment or cyclin A1 overexpression alone or in combination on VEGF mRNA level was determined in MCF-7 cells. Cells that were transfected with cyclin A1pCMS-EGFP or pCMS-EGFP vectors in the absence or presence of E2 are indicated. (C) Evaluation of the effects of Tamoxifen (Tam), E2, and E2 in combination with Tamoxifen (E2+Tam) on VEGF protein expression in MCF-7 cells. MCF-7 cells were transfected with cyclin A1 vector or control vector as indicated. Data in graphs below are the mean ± SD represents two independent experiments. (D) Immunoblot analysis data obtained in MDA-MB-231 which were treated using the same conditions as mentioned in (C). (E) Immunoprecipitation analysis (IP) shows physical interaction between cyclin A1 and ER-α in MCF-7 cells that were transfected with cyclin A1pCMS-EGFP or pCMS-EGFP vectors. ER-α antibody was used in IP to pull down the immunocomplexes and subsequent Westernblot was performed using cyclin A1 or ER-α antibodies to detect the immunocomplexes as indicated. The input was used as controls as indicated. (F) Evaluation of ER-α mRNA expression in the cells that were transfected with A1pCMS-EGFP or pCMS-EGFP vectors. The representative picture is shown in the upper panel. Quantification of ER-α mRNA level is shown in the lower panel and mean ± SD represents three independent experiments. (G) Western blot analysis shows the expression level of ER-α protein in the cells that were transfected with A1pCMS-EGFP or pCMS-EGFP vectors. Breast cancer cell lines used for these studies are T47D, MCF-7 and MDA-MB231 as indicated.

Next, we investigated whether there may be a functional link between cyclin A1 and estrogen signalling. We treated MCF-7, MDA-MB-231 and T47D cells with E2 and determined the effect of E2 on cyclin A1 and VEGF expression. Treatment of MCF-7 and T47D cells with E2 increased VEGF mRNA expression, but E2 stimulation did not shown apparent effect on VEGF expression in MDA-MB-231 cells (Figure 4A), thus suggesting that E2 stimulation influences VEGF expression only in ER-α positive breast cancer cells but not in MDA-MB-231 cells that lack ER-α expression. Our findings are in agreement with previous reported studies [7,14]. We also observed that E2 treatment resulted in a slight increase in cyclin A1 mRNA in MCF-7 and T47D cells (Figure 4A). Based on these findings, we next investigated whether cyclin A1, ER and VEGF are functionally coordinated or overlapped with each other. To this end, we firstly assessed whether cyclin A1 overexpression together with E2 stimulation may have additive effect on VEGF expression in MCF-7 cells. MCF-7 cells transfected with cyclin A1-pCMS-EGFP or control pCMS-EGFP vectors were treated with E2 or solvent. VEGF expression was much higher in MCF-7 cells transfected with cyclin A1-pCMS-EGFP and were treated with E2 compared with the cells transfected with pCMS-EGFP alone or treated with E2 alone (Figure 4B). These data suggest that cyclin A1 overexpression along with E2 stimulation have additive effect on VEGF expression in MCF-7 cells. We next assessed whether inhibition of the levels of estrogen and ER-α expression may reduce VEGF expression which was initially induced by estrogen and cyclin A1 overexpression. MCF-7 cells transfected with cyclin A1-pCMS-EGFP or pCMS-EGFP vectors were treated with tamoxifen alone, estrogen alone, or estrogen and tamxifenin in combination, and VEGF expression was then examined in these cells. Treatment of MCF-7 cells expressing control pCMS-EGFP vector with tamoxifen alone led to an increase in VEGF expression (Figure 4C), which was consistent with the reported studies where tamoxifen treatment induced VEGF expression in MCF-7 cells [41]. Treatment of MCF-7 cells overexpressing cyclin A1-pCMS-EGFP with tamoxifin did not lead to an increase in VEGF expression (Figure 4C). Since the overall VEGF protein expression was much higher in MCF-7 cells transfected with cyclin A1-pCMS-EGFP than the cells transfected with pCMS-EGFP (Figure 4C). Treatment of these cells with E2 resulted in a more pronounced increase in VEGF expression in MCF-7 cells transfected with cyclin A1-pCMS-EGFP (Figure 4C). As tamoxifen inhibits E2 effect in ER-α positive breast cancer cells by interfering ER-α, we then examined the effect of E2 and tamoxifin combination treatment on MCF-7 cells transfected with cyclin A1-pCMS-EGFP or pCMS-EGFP. VEGF expression was reduced in MCF-7 cells transfected with cyclin A1-pCMS-EGFP or pCMS-EGFP, which were treated with E2 and tamoxifin in combination, compared with the cells treated with E2 alone (Figure 4C). Strikingly, in MDA-MB-231 cells that lack ER-α expression VEGF expression was not influenced by cyclin A1 overexpression alone, or E2 treatment alone or E2 and tamoxifen and cyclin A1 overexpression in combination (Figure 4D). Taken together, these data show that inhibition of E2 and ER-α by tamoxifen reduces VEGF expression which was initially induced by cyclin A1 overexpression and E2 stimulation. These data suggest that cyclin A1, estrogen and VEGF pathways are functionally linked and overlap with each other in breast cancer cells. Next, we examined the relationship between cyclin A1 and ER-α. To this end, we investigated whether cyclin A1 is present in ER-α associated immunocomplexes or *vice versa* in MCF-7 cells. MCF-7 cells were transfected with cyclin A1-pCMS-EGFP or pCMS-EGFP and were subjected to immunoprecipitation analysis. There was indeed a formation of protein–protein complexes between cyclin A1 and ER-α in MCF-7 cells (Figure 4E), and in MCF-7 cells transfected with cyclin A1-pCMS-EGFP (Figure 4E and Figure S3).

To investigate whether induced cyclin A1 overexpression may lead to alterations in ER-α expression in breast cancer cells, we examined ER-α expression in MCF-7, MDA-MB231 and T47D cells which were transfected with cyclin A1-pCMS-EGFP or pCMS-EGFP vectors. RT-PCR and immunoblot analysis showed that ER-α mRNA and protein expression were increased in MCF-7 cells transfected with cyclin A1-pCMS-EGFP compared with the cells transfected with pCMS-EGFP (Figure 4F and G). This data suggests that overexpression of cyclin A1 results in an increase in ER-α expression in MCF-7 cells. ER-α expression was undetectable in MDA-MB-231 cells transfected with cyclin A1-pCMS-EGFP or pCMS-EGFP vectors (Figure 4F and G), which confirmed that MDA-MB-231 cells did not express ER-α.

### Overexpression of Cyclin A1 promoted tumor growth and vascularization in animal model

**Figure 5:**
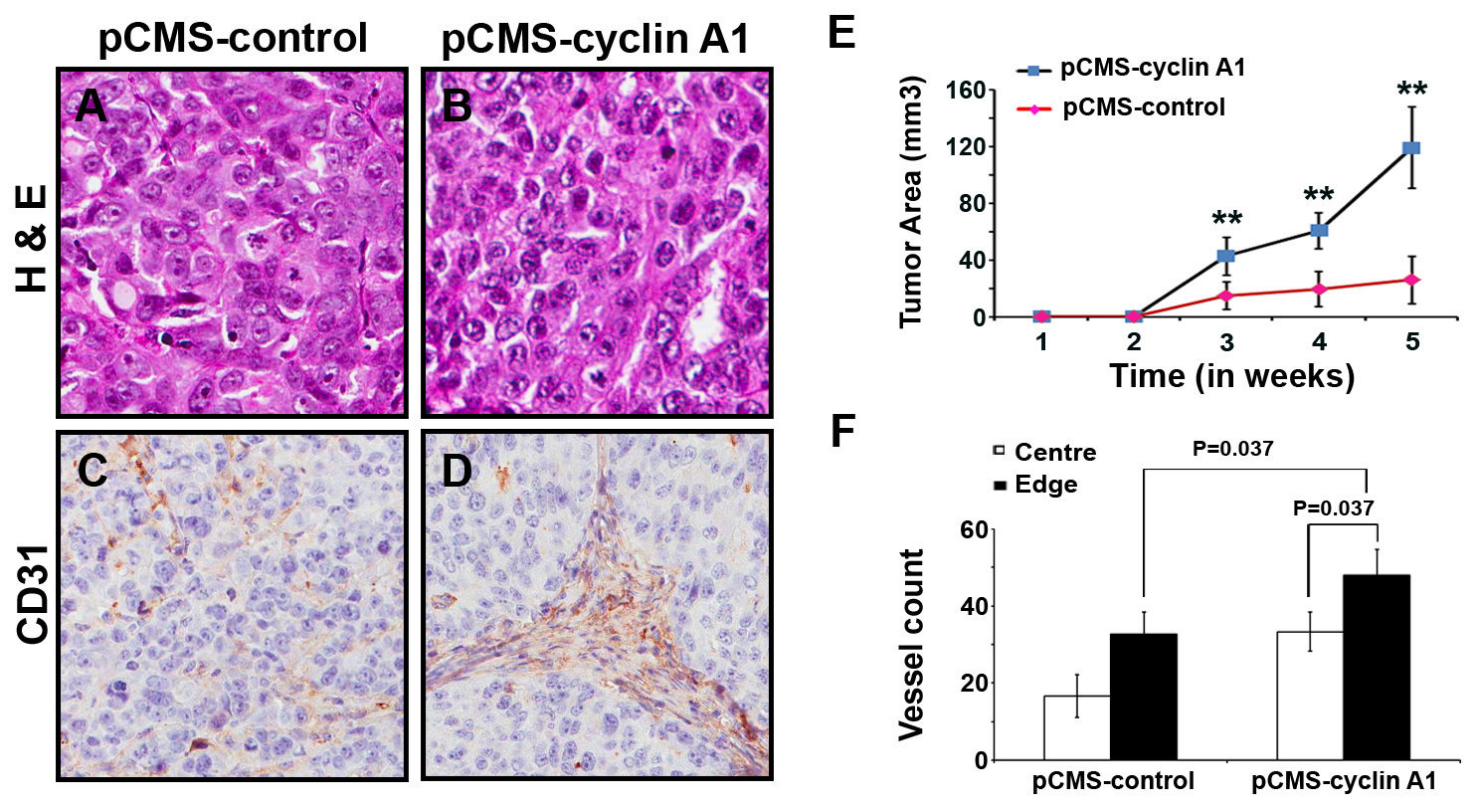
The effect of cyclin A1 on growth and vascularization of tumor xenografts *in mice.* MCF-7 cells transfected with cyclin A1pCMS–EGFP or pCMS-EGFP vectors were subcutaneous implanted into female nude mice with E2 supplementation. (A, B) Representative microphotographs of xenograft tumor sections stained with Haematoxylin and Eosin are shown. The control tumor “pCMS-control” and cyclin A1 expressing tumor “pCMS-cyclin A1” are indicated. (C, D) Representative pictures show the xenograft tumors stained with antibody against human CD31, the CD31 positive vessels are indicated. (E) Growth curves of the two groups of xenograft tumors. The control tumor “pCMS-control” and cyclin A1 expressing tumor “pCMS-cyclin A1” are indicated. The time is indicated in x-axis and tumor volume in mm^3^ is indicated in y-axis. (F) Quantification of the tumor vascularizations in cyclin A1 expressing xenograft tumors “pCMS-cyclin A1” and in control xenograft tumors “pCMS-control”. The numbers of CD31-positive blood vessels in the central vs. edge regions of the tumor areas are shown. P values are indicated. Mean ± SD represents three independent experiments.

Based on our observations in primary breast cancer specimens and metastatic lesions, and our data from functional analysis in breast cancer cell lines, we hypothesized that altered cyclin A1 expression may contribute to breast cancer progression in cooperation with VEGF and ER-α. To elucidate biological consequences of cyclin A1 overexpression in breast cancer cells, we established mouse tumor xenograft models in which we implanted subcutaneously MCF-7 cells transfected with cyclin A1-pCMS-EGFP or pCMS-EGFP vectors into female nude mice. Tumor growth in xenograft mice were monitored for 5 weeks and the tumors were collected at the end of experiments. The tumor histology was examined and angiogenesis of each tumor was measured by determining the numbers and densities of CD-31-positive blood vessels (Figures 5A, 5B, 5C and 5D) as described previously [33]. At the end of experiments, tumors that overexpressed cyclin A1 were significantly larger, with a mean tumor volume of 119±28 mm^3^, compared with the tumors that expressed control vector, with a mean size 25.9±17 mm^3^ (*p*<0.01) (Figure 5E). Tumors that expressed cyclin A1 displayed significantly increase in the number of CD31-positive vessels in both central and edge areas compared with that of the controls (p=0.037) (Figure 5F). We further observed that VEGFR1 expression was in general higher in both endothelial cells of blood vessels and tumor cells from tumors that overexpressed cyclin A1 (Figure S4). These data suggest that induction of cyclin A1 overexpression in breast cancer cells results in an increase in tumor growth and angiogenesis in xenograft mouse model.

### Overexpression of cyclin A1 promoted tumor growth and VEGF signaling in tumor xenografts bearing tumors stably expressing cyclin A1

**Figure 6:**
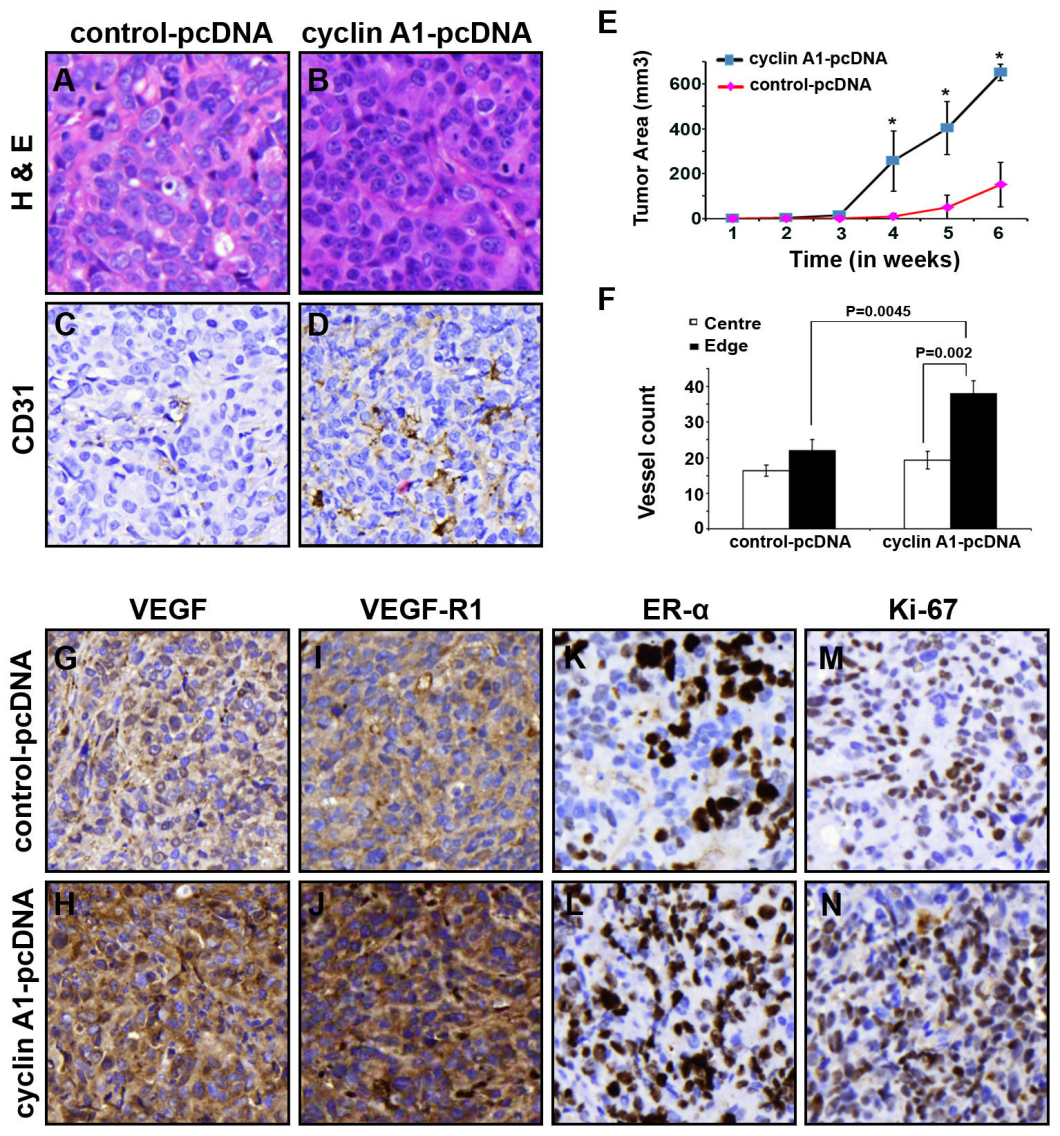
Long-term effect of elevated level of cyclin A1 on growth and angiogenesis phenotype of xenograft tumors *in mice.* MCF-7 cells stable expressing pcDNA–cyclin A1 or pcDNA vectors were subcutaneous implanted into female nude mice with E2 supplementation. (A, B) Representative microphotographs of xenograft tumor sections stained with Haematoxylin and Eosin are shown. (C, D) Representative pictures show the xenograft tumors stained with antibody against human CD31, the CD31 positive vessels are indicated. The control tumor “control-pcDNA” and cyclin A1 expressing tumors “cyclin A1-pcDNA” are indicated. (E) Growth curves of the two groups of xenograft tumors are indicated. The time is indicated in x-axis and tumor volume in mm^3^ is indicated in y-axis. (F) Quantification of the tumor vascularizations in cyclin A1 expressing xenograft tumors “cyclin A1-pcDNA” and in control xenograft tumors “control-pcDNA”. The numbers of CD31-positive blood vessels in the central vs. edge regions of the tumor areas are shown. P values are indicated. Mean ± SD represents three independent experiments. (G–N) Xenograft tumors from “cyclin A1-pcDNA” and “control-pcDNA” groups were immunostained with antibodies against VEGF, VEGFR1, ER-α and Ki67. The representative microphotographs are shown.

To further validate the role of cyclin A1 and its functional link with VEGF and ER-α in tumor growth and angiogenesis, we generated MCF-7 cells that were stably transfected with cyclin A1-pcDNA or control-pcDNA vector. The constitutively high level of cyclin A1 expression in MCF-7 cells was achieved. We then established xenograft mice in which a larger number of tumor cells which constitutively overexpressed cyclin A1-pcDNA or control-pcDNA vectors were subcutaneously implanted into mice. The growth of tumors in mice was monitored for 6-weeks, and the tumors were collected for evaluation of histology. An infiltrative growth pattern in the fibroblast capsule areas was observed in tumors that constitutively overexpressed cyclin A1, but not in the controls (Figure 6A and 6B). The increased CD-31-positive blood vessels were evident in tumors that overexpressed cyclin A1 compared with the controls as determined by evaluating CD-31-stained sections (Figure 6C and 6D). Mice received MCF-7 cells that overexpressed cyclin A1 had significantly larger tumors with a mean size of 650.5±32 mm^3^ compared with controls with a mean size of 150.9±98 mm^3^ (p=0.02) (Figure 6E). CD31-positive vessels at central and edge areas of tumors were significantly higher in xenograft tumors that overexpressed cyclin A1 compared with that of controls (p<0.01) (Figure 6F). These data suggest that constitutively overexpression of cyclin A1 promoted growth and vascularization of breast tumors in xenograft mice. Because increased vascularization and tumor growth are often due to increased activity of VEGF signalling, we subjected tumors collected from xenograft mice to immunohistochemical analysis using antibodies against VEGF, VEGFR1. The increased expression of VEGF and VEGFR1 was observed in tumors that overexpressed cyclin A1 (Figures 6G, 6H, 6I, 6J). There was a higher proportion of ER-α positive tumor cells in xenograft tumors that overexpressed cyclin A1 compared with the tumors expressed control vector (Figure 6K and 6L). The proliferative activities in the tumors were also measured by quantifying the fraction of Ki-67 positive tumor cells. The proportion of Ki-67 positive cells was increased in xenograft tumors that overexpressed cyclin A1 (Figure 6M and 6N). These data suggest that induced overexpression of cyclin A1 enhanced expression of VEGF, ER-α and Ki-67. The enhanced expression of VEGF, ER-α and Ki-67 which are induced by cyclin A1 overexpression may contribute to the growth and angiogenesis of xenograft tumors in mice. Cyclin A1 therefore plays an important role in breast cancer progression by influencing both VEGF and ER-α pathways.

## Discussion

The majority of normal human tissues express very low level of cyclin A1 transcript as reported in several previous studies [21–24]. In the present study, we show that cyclin A1 mRNA is expressed in normal breast tissue at very low level, which is consistent with the reported studies. We further show that cyclin A1 is highly expressed in primary breast cancer specimens and metastatic lesions from breast cancer patients. There is a statistically significant correlation between cyclin A1 and VEGF expression in the cancer specimens from the two patient cohorts. To our knowledge, the current study is the first to describe cyclin A1 expression in large clinical materials including primary cancer and metastatic lesions from patients with breast cancer. Thus our study suggests a clinical importance of cyclin A1 expression in breast cancer. The functional consequences of cyclin A1 overexpression have been elucidated in leukemic and prostate cancer cell lines and mouse models [32-33]. These previous findings demonstrate that abnormal expression of cyclin A1 results in the development of leukemia and progression of prostate cancer [32-33]. In the present study, we investigate a role of cyclin A1 in breast cancer progression by employing breast cancer cell lines and xenograft mouse models. We show that induction of cyclin A1 overexpression in MCF-7 and MDA-MB-231 cells results in an increase in the invasiveness of these cells. This finding is of particular interesting, as overexpression of cyclin A1 has been shown to promote invasion of prostate cancer cells in orthotopic xenograft mouse model [33]. Our findings suggest that cyclin A1 is involved in breast cancer progression in addition to its role in the initiation of leukemia and prostate cancer.

To further understand the mechanisms by which cyclin A1 promotes the invasiveness of breast cancer cells, we investigate whether cyclin A1 is functionally linked to VEGF by using breast cancer cell lines that are either positive or negative for ER-α expression. We show that induction of cyclin A1 overexpression in MCF-7 cells leads to an increase in VEGF mRNA and protein expression, which is coincident with the increased invasiveness of these cells. As elevated level of VEGF signalling may enhance angiogenesis and promote tumor cell invasion, the altered VEGF expression in MCF-7 cells that overexpress cyclin A1 may contribute to tumor cell invasion.

In the present study, we show that cyclin A1 is functionally linked to ER-α in MCF-7 cells by forming a protein–protein complexes with ER-α. Induction of cyclin A1 expression leads to an increase in ER-α expression in MCF-7 and T47D cells which are estrogen responsive breast cancer cells. It is known that ER-α−mediated cellular pathways are involved in proliferation and angiogenesis of breast cancer cells [5]. Estrogen stimulation increases VEGF mRNA expression, and estrogen exerts its effect on VEGF via ER-α [5,14,15]. In the present study, we discover that E2 treatment and cyclin A1 overexpression have additive effect on VEGF expression in MCF-7 cells. We further show that inhibition of estrogen by tamoxifen reduces VEGF expression which is initially induced by E2 and cyclin A1 overexpression in MCF-7 cells. Our data suggest that altered cyclin A1 expression may mediate responsiveness of MCF-7 cells to E2 by modulating the expression levels of VEGF and ER-α. Our data show that cyclin A1 modulates VEGF expression in MCF-7 cells but not in MDA-MB-231 cells, as MDA-MB-231 cells lack ER-α expression, and given that cyclin A1 is physically and functionally associated with ER-α, it is likely that cyclin A1 modulates VEGF expression in part through ER-α inMCF-7 cells. Because tamoxifen treatment of MCF-7 cells that overexpressed cyclin A1 did not completely inhibit VEGF expression which was initially induced by cyclin A1 overexpression, this data suggests that cyclin A1 may modulates VEGF expression through additional pathways that are independent of ER-α. We have previously shown that altered cyclin A1 expression promotes prostate cancer metastasis by inducing the expression and activities of MMPs in prostate cancer cells [33]. It will be of interesting to investigate whether MMPs may be involved in mediating invasion of ER-α positive breast cancers in cooperation with cyclin A1, ER-α and VEGF. Increased VEGF expression has previously been shown to impair the response to tamoxifen treatment which is used for clinical treatment of ER-α positive breast cancer [4–6], our finding on that cyclin A1 is involved in the modulation of both ER-α and VEGF pathways will provide novel information for gain better understanding of treatment response.

In our present study, we demonstrate that induction of cyclin A1 overexpression in MCF-7 cells leads to an accelerated growth and angiogenesis of xenograft tumors in mice. MCF-7 is an ER-α positive breast cancer cell line and can only form tumors in the presence of E2 supplementation. We show that cyclin A1 overexpression confers the MCF-7 cells with an increased ability to form larger, highly vascularised tumors in mice. Thus, our finding suggests that cyclin A1 is a potent enhancer of tumorigenesis. It is known that increased VEGF signalling may directly promote growth, invasion and metastasis of breast cancer [41–44]. In addition, VEGFR1 has been shown to enhance angiogenesis by stimulating endothelial cell growth and by recruiting the cytokines from microenvironments [45]. We show that xenograft tumors that overexpress cyclin A1 display increased expression of VEGF and VEGFR1 in the tumor cells and in endothelial cells of blood vessels. Our findings suggest that cyclin A1 may promote invasive tumor phenotypes by recruiting VEGF and VEGFR1 in tumor cells.

It is known that overexpression of co-factor of ER-α results in constitutive activation of ER-α-mediated transcription, and is associated with reduced responsiveness of breast cancer cells to therapy [2]. This could explain the disappointing efficacy of long-term anti-angiogenic or anti-VEGF directed therapies such as bevacizumab on overall survival of late-stage HER2- negative, triple-negative disease. Simultaneous targeting of several key independent yet overlapping pathways may block the ability of breast cancer cells to bypass the inhibitions induced by therapeutic agents and thus improve the likelihood of response to therapy. It is of importance to identify multiple molecular processes that determine of deregulation of proliferation and invasion in endocrine-dependent and independent breast cancer cells. Increased VEGF expression has previously been shown to impair the response to tamoxifen treatment which is used for clinical treatment of ER-α positive breast cancer [4–6]. Given the important role of cyclin A1 and its functional link with ER-α and VEGF which have been identified in the present study, it is of interesting to investigate whether cyclin A1 may play an important in modulating treatment response of breast cancer in laboratory and clinical settings. Our finding on that cyclin A1 is involved in the modulation of both ER-α and VEGF pathways may provide novel insights into treatment of breast cancer and may be helpful for designing novel drugs to selectively target invasive breast cancer cells in clinical settings.

## Supporting Information

Figure S1 **Evaluation of cyclin A1 and VEGF mRNA expression in various types of human normal tissues.** (A) Cyclin A1 mRNA expression in normal breast tissue and various types of normal tissues (n=36) as indicated. (B) VEGF mRNA expression in the same sample settings as mentioned in (A).
(JPG)

Figure S2 **Validation of cyclin A1 overexpression in the breast cancer that were transfected with cyclin A1pCMS-EGFP or pCMS-EGFP vectors.** Cyclin A1 mRNA levels are assessed in T47D, MCF-7 and MDA-MB231 cells after transfection by semiquantitative RT-PCR and a representative picture is shown.
(JPG)

Figure S3 **Immunocomplex formation between cyclin A1 and ER-α.** Immunoprecipitation (IP) assay shows a physical interaction of cyclin A1 with ER-α in MCF-7 cells transfected with cyclin A1pCMS-EGFP or pCMS-EGFP vectors. ER-α antibody was used in IP to pull down ER-α associated immunocomplexes, and the immunoblot analysis was subsequently performed to detect cyclin A1 in ER-α immunocomplexes by using antibody against cyclin A1 as indicated. The input was used as positive control, and IgG without addition of primary antibodies was used as negative control as indicated.
(JPG)

Figure S4 **Evaluation the expression of VEGFR1 in xenograft tumor sections.** The tumor cells and endothelial cells of vessels in control tumor “pCMS-control” and in cyclin A1 expressing tumor “pCMS-cyclin A1” are used for the immunostaining of VEGFR1.
(JPG)

Table S1 **Evaluation of cyclin A1 expression in TMA1 containing cancer specimens from 94 patients with breast cancer.** Cyclin A1 expression was evaluated based on the intensity of the immunostaining using antibody against cyclin A1 in primary breast cancer specimens from 94 patients is summarized as indicated.
(DOC)

Table S2 **Evaluation of cyclin A1and VEGF expression in TMA2 containing cancer specimens from 48 patients with breast cancer.** Cyclin A1 and VEGF expression in primary breast cancer specimens from 48 patients are summarized.
(DOC)

Table S3 **Evaluation of cyclin A1 and VEGF in metastatic lesions.** Cyclin A1 and VEGF expression in tumor cells from lymph node metastasis are summarized.
(DOCX)
