# Supplementary figures and images for: Cyclin A1 Modulates the Expression of Vascular Endothelial Growth Factor and Promotes Hormone-Dependent Growth and Angiogenesis of Breast Cancer
Source: PLoS One. 2013 Aug 8;8(8):e72210. doi: 10.1371/journal.pone.0072210 (PMC3744130; doi:10.1371/journal.pone.0072210)

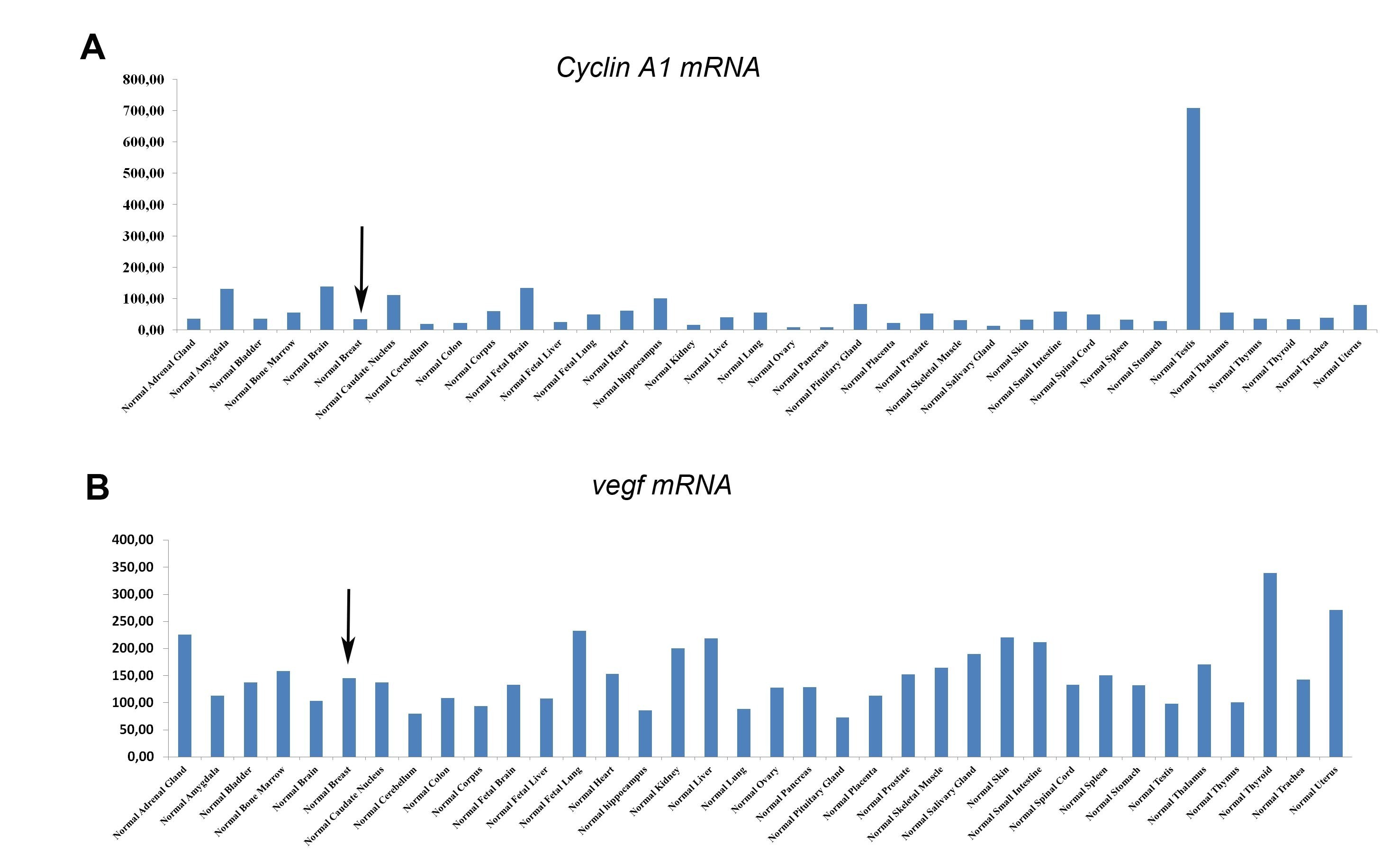

Supplement: Figure S1 — Evaluation of cyclin A1 and VEGF mRNA expression in various types of human normal tissues. (A) Cyclin A1 mRNA expression in normal breast tissue and various types of normal tissues (n=36) as indicated. (B) VEGF mRNA expression in the same sample settings as mentioned in (A). (JPG) [file pone.0072210.s001.jpg]

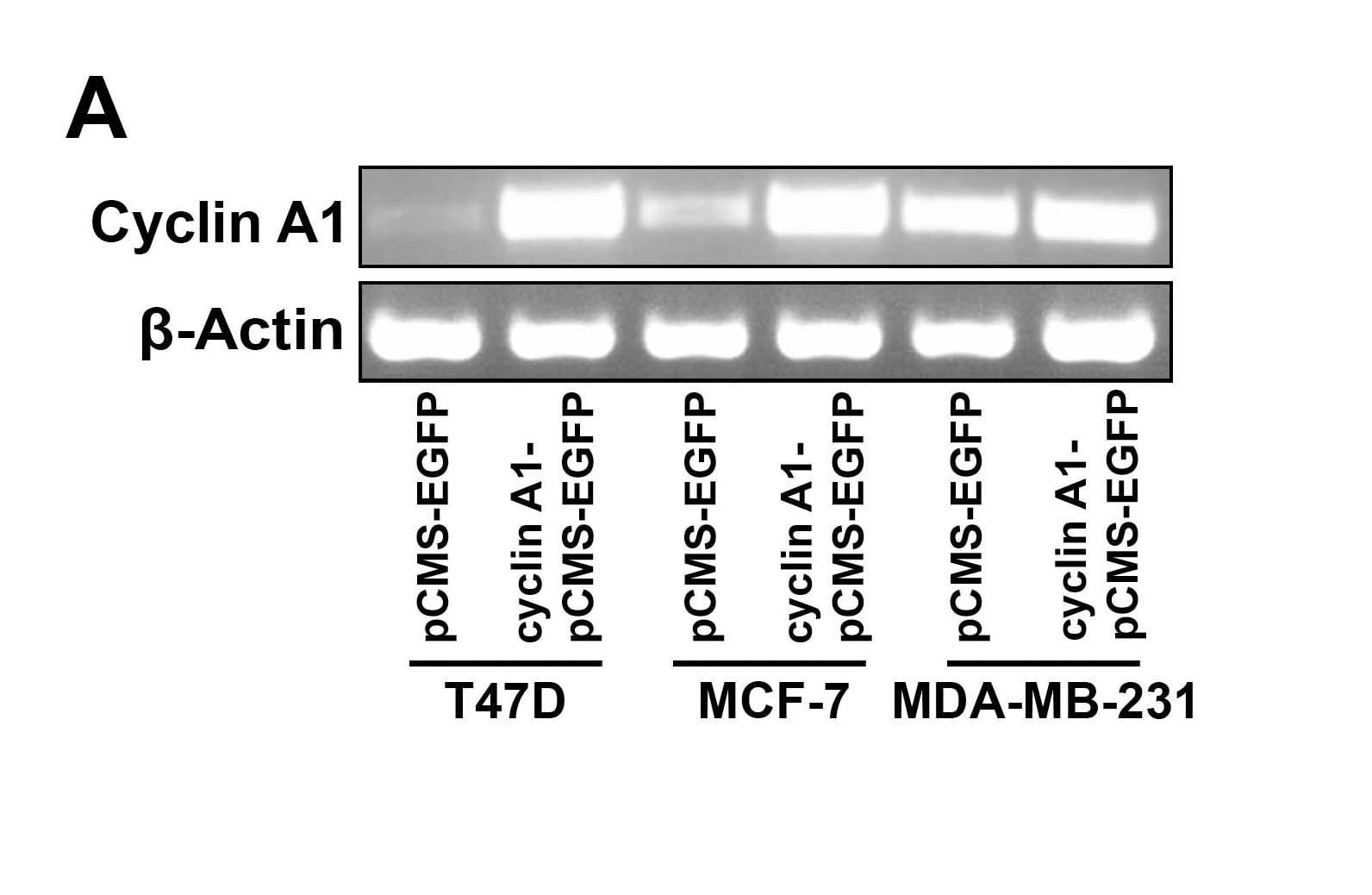

Supplement: Figure S2 — Validation of cyclin A1 overexpression in the breast cancer that were transfected with cyclin A1pCMS-EGFP or pCMS-EGFP vectors. Cyclin A1 mRNA levels are assessed in T47D, MCF-7 and MDA-MB231 cells after transfection by semiquantitative RT-PCR and a representative picture is shown. (JPG) [file pone.0072210.s002.jpg]

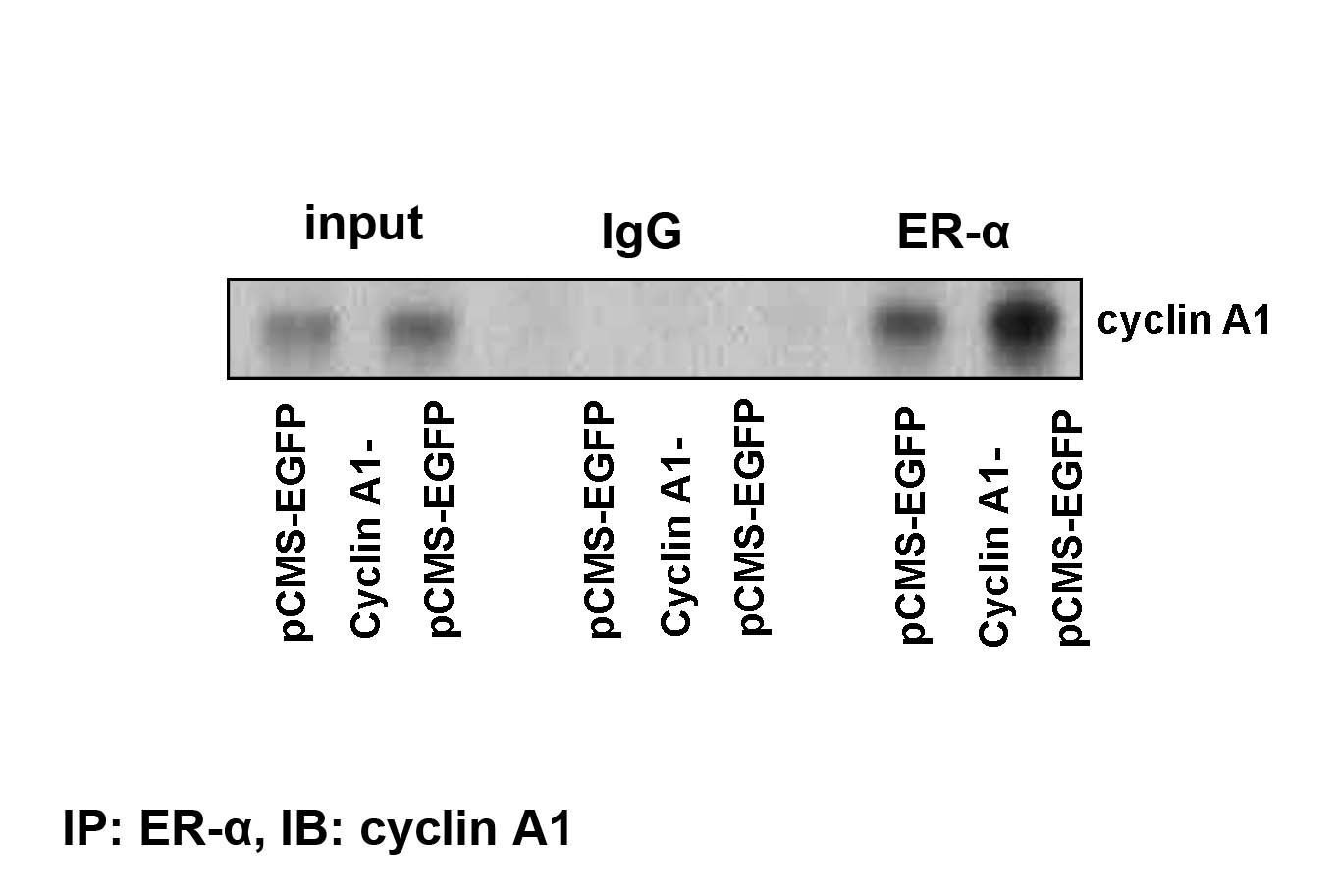

Supplement: Figure S3 — Immunocomplex formation between cyclin A1 and ER-α. Immunoprecipitation (IP) assay shows a physical interaction of cyclin A1 with ER-α in MCF-7 cells transfected with cyclin A1pCMS-EGFP or pCMS-EGFP vectors. ER-α antibody was used in IP to pull down ER-α associated immunocomplexes, and the immunoblot analysis was subsequently performed to detect cyclin A1 in ER-α immunocomplexes by using antibody against cyclin A1 as indicated. The input was used as positive control, and IgG without addition of primary antibodies was used as negative control as indicated. (JPG) [file pone.0072210.s003.jpg]

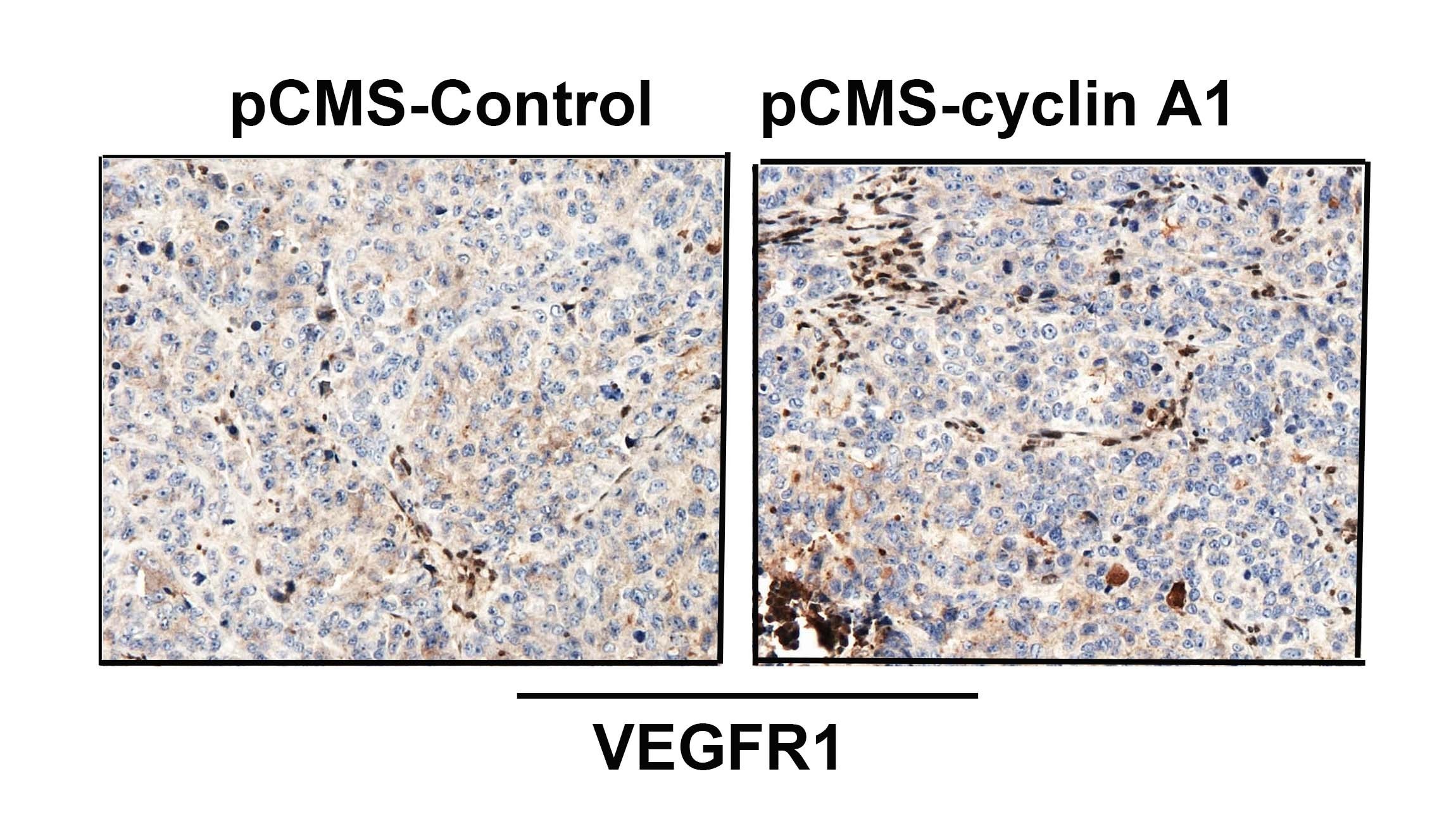

Supplement: Figure S4 — Evaluation the expression of VEGFR1 in xenograft tumor sections. The tumor cells and endothelial cells of vessels in control tumor “pCMS-control” and in cyclin A1 expressing tumor “pCMS-cyclin A1” are used for the immunostaining of VEGFR1. (JPG) [file pone.0072210.s004.jpg]
