# Supplementary material for: Cyclin A1 Modulates the Expression of Vascular Endothelial Growth Factor and Promotes Hormone-Dependent Growth and Angiogenesis of Breast Cancer
Source: PLoS One. 2013 Aug 8;8(8):e72210. doi: 10.1371/journal.pone.0072210 (PMC3744130; doi:10.1371/journal.pone.0072210)
Supplement: Table S1 — Evaluation of cyclin A1 expression in TMA1 containing cancer specimens from 94 patients with breast cancer. Cyclin A1 expression was evaluated based on the intensity of the immunostaining using antibody against cyclin A1 in primary breast cancer specimens from 94 patients is summarized as indicated. (DOC) [file pone.0072210.s005.doc]

**Tabel S1. Evaluation of cyclin A1 expression in TMA1 containing cancer specimens from 94 patients with breast cancer.**

| **Group (scores of cyclin A1 staining intensity)** | **Numbers of patients/specimens:**  **n (%)** |
| --- | --- |
| Low expression (1) | 10 (10.64%) |
| Moderate expression (2) | 32 (34.04%) |
| Strong expression (3) | 52 (55.32%) |
| Total number of samples | 94 (100%) |
