# Supplementary material for: Cyclin A1 Modulates the Expression of Vascular Endothelial Growth Factor and Promotes Hormone-Dependent Growth and Angiogenesis of Breast Cancer
Source: PLoS One. 2013 Aug 8;8(8):e72210. doi: 10.1371/journal.pone.0072210 (PMC3744130; doi:10.1371/journal.pone.0072210)
Supplement: Table S2 — Evaluation of cyclin A1and VEGF expression in TMA2 containing cancer specimens from 48 patients with breast cancer. Cyclin A1 and VEGF expression in primary breast cancer specimens from 48 patients are summarized. (DOC) [file pone.0072210.s006.doc]

**Tabel S2. Evaluation of cyclin A1and VEGF expression in TMA2 containing cancer specimens from 48 patients with brease cancer.**

| ***48 Primary tumors*** | | |
| --- | --- | --- |
| **Cyclin A1 VEGF** | | |
| **Group (scores of staining intensity for cyclin A1 or VEGF)** | **Numbers of specimens:**  **n (%)** | **Numbers of specimens:**  **n (%)** |
| Low expression (1) | 8 (16.67 %) | 9 (18.75 %) |
| Moderate expression (2) | 37 (77.08 %) | 21 (43.75 %) |
| Strong expression (3) | 1 (2.08 %) | 16 (33.33 %) |
| Negative expression (0) | 1 (2.08 %) | 2 (4.17 %) |
| Unscorable (ND) | 1 (2.08 %) | ------ |
| Total number of samples | 48 (100 %) | 48 (100 %) |
