# Supplementary material for: Cyclin A1 Modulates the Expression of Vascular Endothelial Growth Factor and Promotes Hormone-Dependent Growth and Angiogenesis of Breast Cancer
Source: PLoS One. 2013 Aug 8;8(8):e72210. doi: 10.1371/journal.pone.0072210 (PMC3744130; doi:10.1371/journal.pone.0072210)
Supplement: Table S3 — Evaluation of cyclin A1 and VEGF in metastatic lesions. Cyclin A1 and VEGF expression in tumor cells from lymph node metastasis are summarized. (DOCX) [file pone.0072210.s007.docx]

|  | **Cyclin A1** | **VEGF** |
| --- | --- | --- |
| **Group** | **No. of patients (%)** | **No. of patients (%)** |
| Low staining (1) | 11 (30.56%) | 5 (13.89%) |
| Moderate staining (2) | 10 (27.78%) | 11 (30.56%) |
| Strong staining (3) | 13 (36.11%) | 18 (50%) |
| Negative staining (0) | 1 (2.78%) | no |
| Unscorable | 1 (2.78%) | 2 (5.56%) |
| Total number of sampels | 36 (100 %) | 36 (100%) |

**Table S3. Evaluation of cyclin A1 and VEGF in metastatic lesions**
